# DIFFERENCES BETWEEN LIFELONG SINGLES AND PARTNERED INDIVIDUALS IN PERSONALITY TRAITS AND LIFE SATISFACTION

**DOI:** 10.1093/geroni/igae098.0210

**Published:** 2024-12-31

**Authors:** David Richter, Julia Stern, Michael Krämer, Alexander Schumacher, Geoff MacDonald

**Affiliations:** SHARE Berlin Institute, Berlin, Berlin, Germany; University of Bremen, Bremen, Bremen, Germany; University of Zurich, Zurich, Zurich, Switzerland; Munich Research Institute for the Economics of Aging and SHARE Analyses (MEA-SHARE), Munich, Bayern, Germany; University of Toronto, Toronto, Ontario, Canada

## Abstract

Being romantically partnered is widely seen as societal norm, and has been shown to be positively associated with important life outcomes, such as physical and mental health. However, the percentage of singles is steadily increasing, with more people staying single for life. We used the Survey of Health, Ageing and Retirement in Europe (N = 77,064 in retirement age from 27 countries) to investigate the Big Five personality traits and life satisfaction in lifelong singles as compared to (previously) partnered individuals. Specification curve analyses suggested that lifelong singles were less extraverted, conscientious, open to experiences, and less satisfied with their lives. Effects were stronger for never partnered than for never cohabitating or never married individuals, and partly moderated by gender, age, country-level singlehood, and gender ratio. Our study provides insights into characteristics of lifelong singles, and has implications for understanding mental health and structures of social support in older individuals.

